# Non-Homologous End Joining Factors XLF, PAXX and DNA-PKcs Maintain the Neural Stem and Progenitor Cell Population

**DOI:** 10.3390/biom11010020

**Published:** 2020-12-28

**Authors:** Raquel Gago-Fuentes, Valentyn Oksenych

**Affiliations:** 1 Department for Cancer Research and Molecular Medicine (IKOM), Norwegian University of Science and Technology, 7491 Trondheim, Norway; raquel.gago-fuentes@ntnu.no; 2 Department of Circulation and Medical Imaging, Norwegian University of Science and Technology, 7491 Trondheim, Norway; 3 KG Jebsen Centre for B Cell Malignancies, Institute of Clinical Medicine, University of Oslo, 0316 Oslo, Norway; 4 Institute of Clinical Medicine, University of Oslo, 0318 Oslo, Norway

**Keywords:** DNA repair, NHEJ, synthetic lethality, genetic interaction

## Abstract

Non-homologous end-joining (NHEJ) is a major DNA repair pathway in mammalian cells that recognizes, processes and fixes DNA damage throughout the cell cycle and is specifically important for homeostasis of post-mitotic neurons and developing lymphocytes. Neuronal apoptosis increases in the mice lacking NHEJ factors Ku70 and Ku80. Inactivation of other NHEJ genes, either *Xrcc4* or *Lig4*, leads to massive neuronal apoptosis in the central nervous system (CNS) that correlates with embryonic lethality in mice. Inactivation of either *Paxx*, *Mri* or *Dna-pkcs* NHEJ gene results in normal CNS development due to compensatory effects of *Xlf*. Combined inactivation of *Xlf/Paxx*, *Xlf/Mri* and *Xlf/Dna-pkcs*, however, results in late embryonic lethality and high levels of apoptosis in CNS. To determine the impact of NHEJ factors on the early stages of neurodevelopment, we isolated neural stem and progenitor cells from mouse embryos and investigated proliferation, self-renewal and differentiation capacity of these cells lacking either *Xlf*, *Paxx*, *Dna-pkcs*, *Xlf/Paxx* or *Xlf/Dna-pkcs*. We found that XRCC4-like factor (XLF), DNA-dependent protein kinase catalytic subunit (DNA-PKcs) and paralogue of XRCC4 and XLF (PAXX) maintain the neural stem and progenitor cell populations and neurodevelopment in mammals, which is particularly evident in the double knockout models.

## 1. Introduction

Double-strand DNA breaks (DSBs) are common DNA damage events that threaten the stability of our genome. DSBs can be repaired by homologous recombination (HR), classical non-homologous end-joining (C-NHEJ, or simply NHEJ) and alternative end-joining (A-EJ, also known as backup end joining, or microhomology-mediated end joining) [1,2,3,4]. HR is only available during S/G2 cell cycle phases of the cell cycle when the sister chromatid is accessible and then used as a template. C-NHEJ acts throughout the entire cell cycle, sealing directly the broken ends and is the predominant repair pathway in mammalian cells [3,5]. A-EJ is often microhomology-mediated and more obvious in the absence of classical NHEJ [6].

C-NHEJ involves recognition of the DSBs by Ku70/Ku80 heterodimer (Ku), which in turn recruits DNA-dependent protein kinase catalytic subunit (DNA-PKcs) to form a DNA-PK holoenzyme complex that protects free DNA ends. Assembly of DNA-PK triggers the autophosphorylation of DNA-PKcs, as well as DNA-PKcs-dependent phosphorylation of multiple other DNA repair factors [3]. Ku facilitates recruitment of NHEJ proteins, such as X-ray repair cross-complementing factor 4 (XRCC4)-like factor (XLF), a paralogue of XRCC4 and XLF (PAXX), and a modulator of retrovirus infection (MRI). Ligation of the broken ends is performed by the NHEJ factor DNA Ligase 4 (LIG4), which is stabilized by another protein, XRCC4 [1,2,3,4].

Genetic inactivation of *Xrcc4* [7] or *Lig4* [8] in mice results in p53-dependent late embryonic lethality, which correlates with a massive apoptosis in the central nervous system (CNS) [9,10]. Although *Ku70*^−/−^ and *Ku80*^−/−^ knockout mice are viable, they present high levels of apoptosis in CNS and remarkable growth retardation [11,12].

Mice lacking NHEJ factors possess various phenotypes [1,2,3,4]. In particular, *Dna-pkcs*^−/−^ [13], *Xlf*^−/−^ [14,15], *Paxx*^−/−^ [2,16,17,18,19,20] and *Mri*^−/−^ [1,2,21] knockout mice are viable, displaying normal growth, lifespan, and neuronal development. However, inactivation of DNA-PKcs kinase domain (*Dna-pkcs*^KD/KD^) leads to Ku- and p53-dependent embryonic lethality, which correlates with high levels of apoptosis in the CNS [22]. Jiang et al. identified more condensed apoptotic nuclei in the intermediate zone of mouse embryonic brain, suggesting that mutation in *Dna-pkcs* gene affects post-mitotic neurons. The apoptotic neurons were relatively rare in the proliferating ventricular zone [22,23]. Thus, a homozygous D3922A mutation in *Dna-pkcs* resulted in a neurological phenotype similar to observed earlier for mice lacking XRCC4 or LIG4 [7,8,9,10,24], suggesting a defect in the G0/G1 phase of the cell cycle, when NHEJ dominates. Contrary, phosphorylation of DNA-PKcs’ S2038, S2053, S2026, S2050 and S2052 is likely not essential for NHEJ in postmitotic neurons [25]. An impact of DSBs on neural stem and progenitor cells (NSPCs) fitness was recently suggested by using wild type (WT) murine cells and ionizing radiation [26]. While previous studies focused on apoptosis of mature neurons using in vivo NHEJ-deficient mouse models, no attempts were made to address the role of specific NHEJ factors during earlier development stages, e.g., proliferation, self-renewal capacity and differentiation of NSPCs.

More recently, genetic interaction studies uncovered the importance of the NHEJ factors XLF, DNA-PKcs, PAXX and MRI in the development of immune and nervous systems and mouse development in general. Synthetic lethality was reported between *Xlf* and *Dna-pkcs* [20,27,28], then between *Xlf* and *Paxx* [1,2,16,17,19,20], and finally between *Xlf* and *Mri* [1,2,21]. These studies suggested that functions of DNA-PKcs, PAXX, and MRI are partially compensated by XLF. While MRI-deficient NSPCs possessed reduced proliferation and normal self-renewal capacity [29], no similar studies were performed on NSPCs lacking XLF, DNA-PKcs, PAXX, XLF/PAXX or XLF/DNA-PKcs.

Here, using single and double knockout mouse models, we found that XLF, DNA-PKcs and PAXX are required to maintain pluripotency of neural stem cells, including aspects of self-renewal, proliferation, and differentiation to neurons and astrocytes.

## 2. Materials and Methods

### 2.1. Mice

All experimental procedures involving mice were performed according to the protocols approved by the Comparative Medicine Core Facility at Norwegian University of Science and Technology (NTNU, Trondheim, Norway). *Dna-pkcs*^+/−^ [13], *Xlf*^+/−^ [14], and *Paxx*^+/−^ [18] mouse models were previously described. *Dna-pkcs*^+/−^ and *Xlf*^+/−^ mice were imported from Professor Frederick Alt lab (Harvard Medical School and Boston Children’s Hospital, Boston, MA, USA). *Paxx*^+/−^ mice were generated by Oksenych group (NTNU, Trondheim, Norway).

### 2.2. Mouse Genotyping

A conventional polymerase chain reaction (PCR) was used to determine the mouse genotypes. DNA was isolated from ear punches by incubating overnight at 56 °C with 2% proteinase K in DNA lysis solution, containing 10 mM pH = 9.0 Tris, 1 M KCl, 0.4% NP-40 and 0.1% Tween 20. Next, the samples were heat-treated for 30 min at 95 °C. The PCR reactions were performed using GoTaq^®^G2 Green Master Mix (Promega, WI, USA; #M7823) or Taq 2x Master Mix Kit (New England Biolabs^®^ Inc., Ipswich, MA, USA; #M0270L) according to the manufacturer’s instructions. Each reaction contained 50 ng of DNA and 0.8 µM of indicated primers (Supplementary Table S5) in a final volume of 25 μL. The PCR product was revealed in a 0.7% agarose gel.

### 2.3. Neural Stem and Progenitor Cell Cultures

NSPCs were cultured as free-floating aggregates, also known as neurospheres [29,30]. Briefly, murine embryos were collected at embryonic day E15.5, the brains were isolated and the cerebellums were removed. Remaining brain parts were mechanically disrupted in proliferation medium, containing Dulbecco Modified Eagle Medium/Nutrient Mixture F12 (DMEM/F12), supplemented with 1% penicillin/streptomycin, 2% B27 without vitamin A (Thermo Fischer Scientific, Waltham, MA, USA), 10 ng/mL epidermal growth factor (EGF) and 20 ng/mL basic fibroblast growth factor (bFGF). The neurospheres were formed and incubated at 37 °C, 5% CO_2_ and 95% humidity. The neurospheres were dissociated every seventh day using 0.25% of trypsin in ethylenediaminetetraacetic acid (EDTA), as previously described in Castaneda-Zegarra et al. (2019) and Wang et al. (2010) [29,30]. For more details, see also Supplementary Materials and Methods. The neurospheres from passages 3 to 10 were used in all the experiments.

### 2.4. Proliferation Assay

NSPCs’ proliferation rates were analyzed using PrestoBlue™ Cell Viability Assay (Thermo Fisher Scientific, Waltham, MA, USA; A13261) following the manufacturer’s protocol and as described in Xing and Oksenych (2019) [31]. Briefly, 0.25% trypsin-EDTA was used to obtain single NSPCs, which were then plated onto a 96-well-suspension plate at 8000 cells per well in proliferation medium and incubated at 37 °C, 5% CO_2_ and 95% humidity. At day 3, PrestoBlue™ was added to final concentration of 10% in proliferation medium in each well, and the cells were incubated for 2 h at 37 °C, 5% CO_2_ and 95% humidity before measuring the fluorescence intensity using FLUOstar Omega system (BMG Labtech, Ortenberg, Germany), 570 nm. The fluorescence intensity indicates the proportion of live cells. The NSPC proliferation assay was carried out on 6 replicates per clone, in 3 independent experiments.

### 2.5. Self-Renewal Capacity Assay

For self-renewal assay, we followed the protocol described earlier [29]. Briefly, the capacity of neural stem cells to maintain their multipotency ex vivo was assessed by determining the number and two-dimensional size of neurospheres. Dissociated single NSPCs were plated onto 6-well suspension plates containing proliferation medium (day 0). At day 8, images of the entire wells were captured using the EVOS microscope (Invitrogen, Carlsbad, CA, USA). The pictures were analyzed using the ImageJ software (NIH, Bethesda, MD, USA) to obtain the total number of neurospheres per well and size of spheres (pixels, px).

### 2.6. Differentiation Assay

Differentiation was induced in dissociated NSPCs, as described previously [29,30]. Briefly, 25,000 single NSPCs were cultured onto 48-well plates pre-coated with 30 µg/mL poly-D-lysine and 2 µg/mL laminin, with differentiation medium containing NeuroBasal A medium (Thermo Fischer Scientific, USA) supplemented with 1% penicillin/streptomycin, 2% B27, 1% GlutaMAX and 10 ng/mL bFGF (day 0) (also see Supplementary Materials and Methods). On day 5, the differentiated cells were fixed with 4% paraformaldehyde for 15 min at room temperature. Furthermore, immunostaining was performed using antibodies recognizing either the neuron-specific β-III tubulin (Tuj1) or the glial fibrillary acidic protein (GFAP) proteins, to determine neurons and astrocytes respectively after differentiation [29,30]. Briefly, the cells were permeablized with 0.1% Triton X-100 for 30 min, washed 3 times with phosphate-buffered saline (PBS) (Oxoid Limited, Basingstoke, UK), and blocked with 1:2 dilution of blocking solution containing 10% BSA (Sigma, USA), 10% goat serum (Invitrogen, USA) and 0.1% Triton X-100 (Sigma, USA) for an hour, and washed with PBS. Then, the cells were incubated with the indicated primary antibodies in 10% blocking solution for one hour at room temperature and washed with PBS. Next, the cells were incubated for one hour with the secondary fluorescent marker-conjugated antibodies at room temperature and counterstained with 1 µg/mL of 4′6-diamidino-2-phenylindole (DAPI, Molecular Probes, Eugene, OR, USA). Images were collected using the EVOS microscope. Positively-stained cells were counted using ImageJ software and presented as a proportion of total cells normalized to WT control.

### 2.7. Western Blot

Western blots were performed using antibodies against XLF, PAXX, DNA-PKcs, and β-actin (Supplementary Materials and Methods) [18,31,32]. Neurospheres were collected and lysed with radioimmunoprecipitation assay buffer (RIPA, Sigma, USA) containing cOmplete™ EDTA-free Protease Inhibitor Cocktail (Roche, Branchburg, NJ, USA) and 1 mM phenylmethanesulfonyl fluoride (PMSF, Sigma, USA). Protein concentrations were determined by Bradford assay (Biorad, Hercules, CA, USA). Further, 40 µg of protein from each clone was analyzed by the sodium dodecyl sulfate-polyacrylamide gel electrophoresis (SDS-PAGE). Proteins were transferred to the membranes using XCell II™ Blot Module (ThermoFisher Scientific, Waltham, MA, USA) at 4 °C. Then, the membranes were blocked with 5% milk in PBS with 10% Tween 20 (PBST) for one hour at room temperature. Primary antibodies were incubated overnight at 4 °C, rinsed with PBST 3 times for 5 min and incubated with the secondary antibodies for one hour at room temperature. The blot was washed and incubated with SuperSignal™ West Femto (Thermo Fischer Scientific, USA) to reveal the proteins with ChemiDoc™ Touch Imaging System (BioRad, USA).

### 2.8. Statistical Analysis

To analyze the data, we pulled together two clones per genotype, representing an independent mouse embryo each, and performed three independent experiments with every clone. All the data shown were normalized to WT average levels. To find statistical differences among the genotypes, Kruskal-Wallis test with Dunn’s multiple comparisons test, as a non-parametric alternative of one-way ANOVA, was used. The statistical analyses were performed using GraphPad Prism 7.03 software (GraphPad Prism, La Jolla, CA, USA) [29,30].

## 3. Results

### 3.1. Impact of XLF, PAXX, and DNA-PKcs on Proliferation and Self-Renewal Capacity of Neural Stem and Progenitor Cells

**Figure 1:**
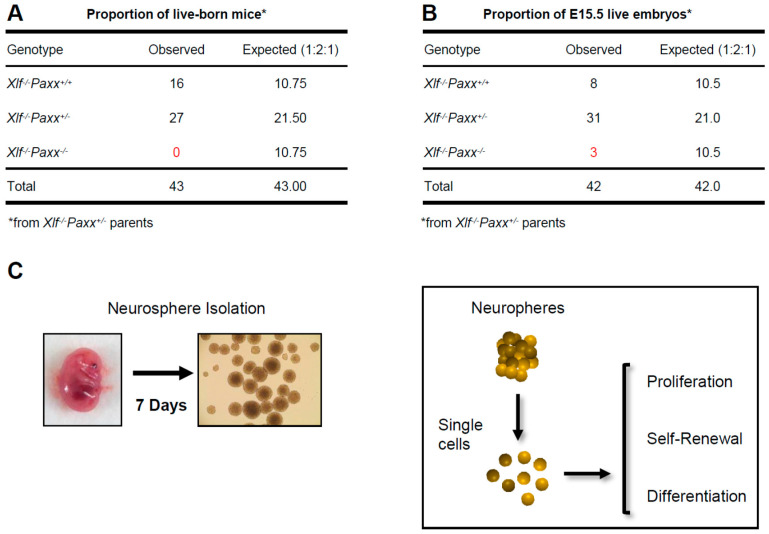
Workflow of the neurosphere-based experiments. (**A**) Synthetic lethality between *Xlf* and *Paxx* in mice. The proportion of live-born mice from *Xlf*^−/−^*Paxx*^+/−^ parents. No *Xlf*^−/−^*Paxx*^−/−^ double knockout live-born mice were observed out of 43 pups analyzed. (**B**) Fifteen-day-old *Xlf*^−/−^*Paxx*^−/−^ mouse embryos are alive. The proportion of genotypes from *Xlf*^−/−^*Paxx*^+/−^ parents. Three E15.5 *Xlf*^−/−^*Paxx*^−/−^ embryos were detected out of 42 analyzed. (**C**) Schematic view of the experiment. Embryos were collected at day E15.5 and neural stem and progenitor cells (NSPCs) were isolated from the embryonic brains. Single NSPCs formed neurospheres in cell culture. Every seventh day, the neurospheres were treated with trypsin to obtain NSPCs used to perform the proliferation, self-renewal and differentiation experiments.

Single knockout of NHEJ genes *Xlf*, *Dna-pkcs* or *Paxx* results in viable fertile mice without detectable phenotypes in the CNS [13,14,15,16,17,18,19]. Contrary, combined inactivation of *Xlf* and *Dna-pkcs* [20,27,28], or *Xlf* and *Paxx* [2,16,17,19,20] results in a synthetic lethality (Figure 1A). To further investigate the impact of XLF, DNA-PKcs, and PAXX on the nervous system development, we isolated NSPCs from WT, *Xlf*^−/−^, *Paxx*^−/−^, *Dna-pkcs*^−/−^, *Xlf*^−/−^*Paxx*^−/−^, and *Xlf*^−/−^*Dna-pkcs*^−/−^ mouse embryos (E15.5). NSPCs aggregated themselves form neurospheres within 7 days in culture. We used these neurospheres to characterize proliferation, self-renewal, and neural differentiation capacity of the NSPCs (Figure 1C).

**Figure 2:**
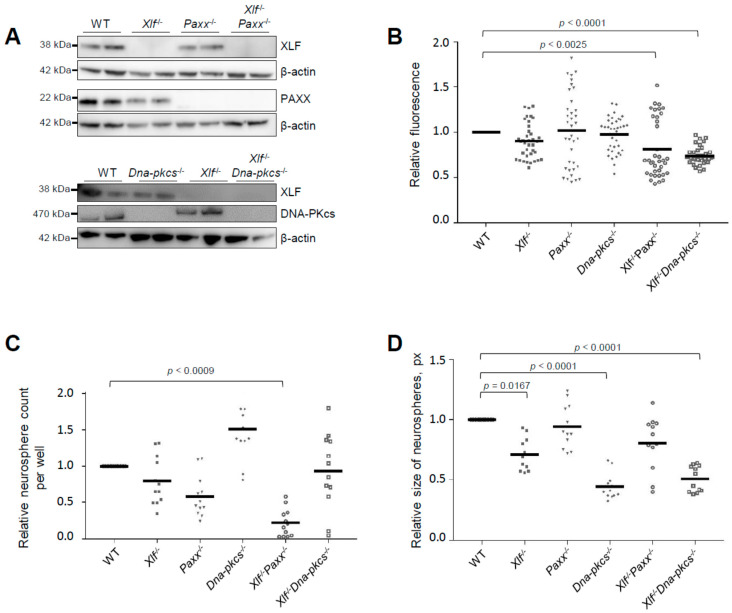
Proliferation and self-renewal capacity of NSPCs. (**A**) Western blot analysis confirmed the NSPCs’ genotype revealing no signal corresponding to XRCC4-like factor (XLF) in *Xlf*^−/−^, *Xlf*^−/−^*Paxx*^−/−^ and *Xlf*^−/−^*Dna-pkcs*^−/−^ NSPCs; no signal corresponding to PAXX in *Paxx*^−/−^ and *Xlf*^−/−^*Paxx*^−/−^ NSPCs; no signal corresponding to DNA-PKcs in *Dna-pkcs*^−/−^ and *Xlf*^−/−^*Dna-pkcs*^−/−^ NSPCs; β-actin was used as a loading control. Uncropped western blots are presented in Supplementary Figure S1. (**B**) Proliferation of NSPCs of indicated genotypes was assessed following 3 days in cell culture; it was expressed as fluorescence units normalized to wild type (WT) controls. Summary of six replicates per two clones, where each clone represents an independent mouse embryo, and three independent experiments (total *n* = 36). The horizontal bars represent the average. Significant differences were found between WT vs. *Dna-pkcs*^−/−^ and WT vs. *Xlf*^−/−^*Paxx*^−/−^. Self-renewal capacity was determined at day 8 in culture by counting the number of neurospheres formed (**C**). Self-renewal was also assessed by (**D**) the size of the neurospheres was counted using pixels (px). Significant differences were found between WT vs. *Xlf*^−/−^ as well as between *Dna-pkcs*^−/−^ vs. *Xlf*^−/−^*Dna-pkcs*^−/−^ samples. Summary of two replicates per clone, two clones per genotype representing an independent mouse embryo each; three independent experiments (*n* = 12). The horizontal bars represent the average values.

To obtain *Xlf*^−/−^*Paxx*^−/−^ embryos, we intercrossed *Xlf*^−/−^*Paxx*^+/−^ mice. As we observed previously [20], no live-born *Xlf*^−/−^*Paxx*^−/−^ pups were detected (0), while we recorded *Xlf*^−/−^*Paxx*^+/+^ (16) and *Xlf*^−/−^*Paxx*^+/−^ (27) live-born mice (Figure 1A). By analyzing E15.5 embryos in the same breedings, we detected *Xlf*^−/−^*Paxx*^−/−^ (3), *Xlf*^−/−^*Paxx*^+/+^ (8) and *Xlf*^−/−^*Paxx*^+/−^ (31) mice (Figure 1B), which were later used for the neurosphere generation and characterization. *Xlf*^−/−^*Dna-pkcs*^−/−^ mice were described earlier [20]. Briefly, by breading *Xlf*^−/−^*Dna-pkcs*^+/−^ mice, we obtained no adult *Xlf*^−/−^*Dna-pkcs*^−/−^ mice (0), while there were *Xlf*^−/−^*Dna-pkcs*^+/+^ (35) and *Xlf*^−/−^*Dna-pkcs*^+/−^ (54) mice at day P30. However, live-born *Xlf*^−/−^*Dna-pkcs*^−/−^ mice were detected at days P1–2, in line with our previous observations [20,27,28]. We confirmed the lack of protein expression in the knockout cells by western blot (Figure 2A and Supplementary Figure S1).

Detailed statistical analysis for NSPC proliferation, Figure 2B. WT vs. *Xlf*^−/−^ (**, *p* = 0.0085); WT vs. *Paxx*^−/−^ (ns, *p* > 0.9999); WT vs. *Dna-pkcs*^−/−^ (ns, *p* > 0.9999); WT vs. *Xlf*^−/−^*Paxx*^−/−^ (***, *p* < 0.0025); WT vs. *Xlf*^−/−^*Dna-pkcs*^−/−^ (****, *p* < 0.0001); *Xlf*^−/−^ vs. *Paxx*^−/−^ (ns, *p* > 0.9999); *Xlf*^−/−^ vs. *Dna-pkcs*^−/−^ (ns, *p* > 0.9999); *Xlf*^−/−^ vs. *Xlf*^−/−^*Paxx*^−/−^ (ns, *p* > 0.9999); *Xlf*^−/−^ vs. *Xlf*^−/−^*Dna-pkcs*^−/−^ (ns, *p* > 0.9999); *Paxx*^−/−^ vs. *Dna-pkcs*^−/−^ (ns, *p* > 0.9999); *Paxx*^−/−^ vs. *Xlf*^−/−^*Paxx*^−/−^ (ns, *p* = 0.1061); *Paxx*^−/−^ vs. *Xlf*^−/−^*Dna-pkcs*^−/−^ (**, *p* = 0.0015); *Dna-pkcs*^−/−^ vs. *Xlf*^−/−^*Paxx*^−/−^ (**, *p* = 0.0080); *Dna-pkcs*^−/−^ vs. *Xlf*^−/−^*Dna-pkcs*^−/−^ (****, *p* < 0.0001); *Xlf*^−/−^*Paxx*^−/−^ vs. *Xlf*^−/−^*Dna-pkcs*^−/−^ (ns, *p* > 0.9999).

Detailed statistical analysis for the relative neurosphere count per well (self-renewal), Figure 2C. WT vs. *Xlf*^−/−^ (*, *p* = 0.0128); WT vs. *Paxx*^−/−^ (***, *p* < 0.0010); WT vs. *Dna-pkcs*^−/−^ (ns, *p* > 0.9999); WT vs. *Xlf*^−/−^*Paxx*^−/−^ (**, *p* < 0.0009); WT vs. *Xlf*^−/−^*Dna-pkcs*^−/−^ (ns, *p* > 0.9999); *Xlf*^−/−^ vs. *Paxx*^−/−^ (ns, *p* = 0.1557); *Xlf*^−/−^ vs. *Dna-pkcs*^−/−^ (****, *p* < 0.0001); *Xlf*^−/−^ vs. *Xlf*^−/−^*Paxx*^−/−^ (****, *p* < 0.0001); *Xlf*^−/−^ vs. *Xlf*^−/−^*Dna-pkcs*^−/−^ (ns, *p* > 0.9999); *Paxx*^−/−^ vs. *Dna-pkcs*^−/−^ (**, *p* = 0.0064); *Paxx*^−/−^ vs. *Xlf*^−/−^*Paxx*^−/−^ (ns, *p* = 0.9392); *Paxx*^−/−^ vs. *Xlf*^−/−^*Dna-pkcs*^−/−^ (ns, *p* > 0.9999); *Dna-pkcs*^−/−^ vs. *Xlf*^−/−^*Paxx*^−/−^ (****, *p* < 0.0001); *Dna-pkcs*^−/−^ vs. *Xlf*^−/−^*Dna-pkcs*^−/−^ (ns, *p* = 0.7134); *Xlf*^−/−^*Paxx*^−/−^ vs. *Xlf*^−/−^*Dna-pkcs*^−/−^ (**, *p* = 0.0098).

Detailed statistical analysis for the relative size of neurospheres (self-renewal), Figure 2D. WT vs. *Xlf*^−/−^ (*, *p* = 0.0167); WT vs. *Paxx*^−/−^ (ns, *p* > 0.9999); WT vs. *Dna-pkcs*^−/−^ (****, *p* < 0.0001); WT vs. *Xlf*^−/−^*Paxx*^−/−^ (ns, *p* = 0.7434); WT vs. *Xlf*^−/−^*Dna-pkcs*^−/−^ (****, *p* < 0.0001); *Xlf*^−/−^ vs. *Paxx*^−/−^ (**, *p* = 0.0028); *Xlf*^−/−^ vs. *Dna-pkcs*^−/−^ (***, *p* = 0.0009); *Xlf*^−/−^ vs. *Xlf*^−/−^*Paxx*^−/−^ (ns, *p* = 0.1897); *Xlf*^−/−^ vs. *Xlf*^−/−^*Dna-pkcs*^−/−^ (*, *p* = 0.0134); *Paxx*^−/−^ vs. *Dna-pkcs*^−/−^ (***, *p* = 0.0002); *Paxx*^−/−^ vs. *Xlf*^−/−^*Paxx*^−/−^ (ns, *p* > 0.9999); *Paxx*^−/−^ vs. *Xlf*^−/−^*Dna-pkcs*^−/−^ (**, *p* = 0.0033); *Dna-pkcs*^−/−^ vs. *Xlf*^−/−^*Paxx*^−/−^ (*, *p* = 0.0241); *Dna-pkcs*^−/−^ vs. *Xlf*^−/−^*Dna-pkcs*^−/−^ (ns, *p* > 0.9999); *Xlf*^−/−^*Paxx*^−/−^ vs. *Xlf*^−/−^*Dna-pkcs*^−/−^ (ns, *p* > 0.2116).

By analyzing the neurosphere cultures, we observed that the average proliferation rates of *Xlf*^−/−^*Paxx*^−/−^ and *Xlf*^−/−^*Dna-pkcs*^−/−^ double knockout neurospheres were reduced when compared to WT and single-deficient *Xlf*^−/−^, *Dna-pkcs*^−/−^ or *Paxx*^−/−^ neurospheres (Figure 2B). To quantify the self-renewal capacity of neurospheres, we plated 10,000 NSPCs and counted the formed neurospheres at day 8 in culture (Figure 2C). Inactivation of *Xlf* resulted in 20% reduction and inactivation of *Paxx* resulted in a 40% reduction of neurosphere count when compared to WT controls. Combined inactivation of *Xlf* and *Paxx* resulted in about 80% reduction of neurosphere count (Figure 2C), further highlighting the severe neurological phenotype of *Xlf*^−/−^*Paxx*^−/−^ mice observed in vivo [16,17,19]. Surprisingly, inactivation of *Dna-pkcs* resulted in a higher number of viable neurospheres, although of smaller size. Combined inactivation of *Xlf* and *Dna-pkcs* resulted in neurosphere count similar to WT controls. We concluded that inactivation of *Xlf* and *Paxx* affected self-renewal capacity and viability of NSPCs (Figure 2C).

To determine neurosphere growth rate, we used an alternative quantification based on the image size in pixels (px) (Figure 2D). Inactivation of *Xlf*, *Dna-pkcs*, or both *Xlf/Dna-pkcs*, resulted in neurospheres with 30% to 50% reduction in size when compared to WT controls. Inactivation of *Paxx* did not affect the size of neurospheres in WT and *Xlf*-deficient backgrounds (Figure 2D). We concluded that both XLF and DNA-PKcs support growth of NSPCs in neurospheres.

### 3.2. Impact of XLF, PAXX, and DNA-PKcs on Differentiation Capacity of Neural Stem and Progenitor Cells

**Figure 3:**
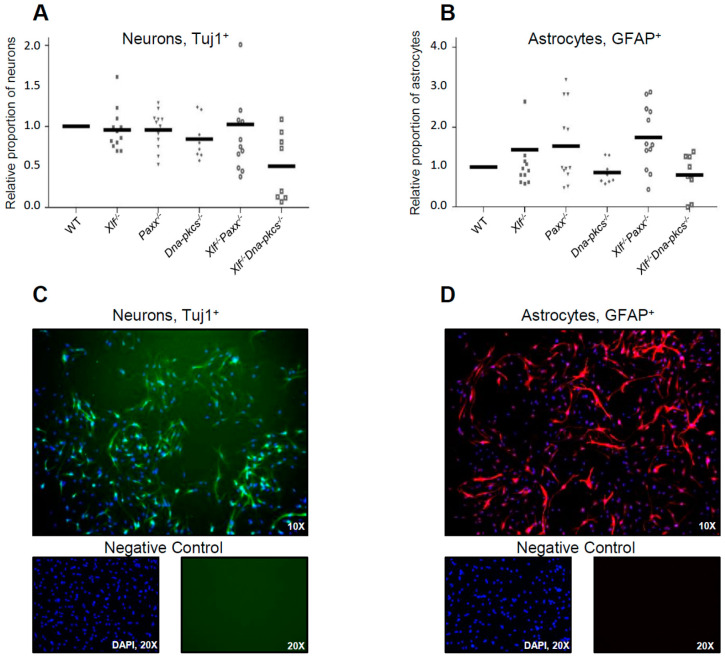
Differentiation of neural progenitors to neurons and astrocytes. (**A**) Proportion of neuron-specific class III beta-tubulin (Tuj1^+^) early neurons and (**B**) Glial fibrillary acidic protein (GFAP^+^) astrocytes following five days of differentiation from NSPCs of indicated genotypes and normalized by WT controls. Average of two replicates per clone, where two clones represent an independent mouse embryo, and three independent experiments (*n* = 12). (**C**,**D**) Examples of the immunostaining using antibodies against Tuj1 and GFAP, as indicated. Tuj1^+^ cells are in green (C). GFAP^+^ cells are in red (**D**). DNA was visualized with 4′,6-diamidino-2-phenylindole (DAPI, blue). Negative controls were performed without adding the primary antibodies (bottom).

To determine whether XLF, PAXX, and DNA-PKcs affect neural differentiation capacity, single NSPCs (25,000 cells) were plated on pre-coated 48-well plates and cultured with differentiation medium for 5 days. Neuronal and glial lineages were identified by immunolabeling using markers for early neurons (Tuj1), and astrocytes (GFAP). Inactivation of *Xlf*, *Paxx* or *Dna-pkcs*, and combined inactivation of *Xlf*/*Paxx* did not affect early neuronal differentiation based on average proportions of Tuj1-positive cells (Figure 3A). Combined inactivation of *Xlf* and *Dna-pkcs*, however, resulted in two-fold reduced neurodifferentiation capacity of NSPCs (Figure 3A,C). The proportion of GFAP-positive glial lineage cells increased, although not significantly, when NSPCs were lacking either XLF or PAXX, or both XLF and PAXX (Figure 3B,D).

Detailed statistical analysis for the neuron differentiation, Figure 3C. WT vs. *Xlf*^−/−^ (ns, *p* > 0.9999); WT vs. *Paxx*^−/−^ (ns, *p* > 0.9999); WT vs. *Dna-pkcs*^−/−^ (ns, *p* > 0.9999); WT vs. *Xlf*^−/−^*Paxx*^−/−^ (ns, *p* > 0.9999); WT vs. *Xlf*^−/−^*Dna-pkcs*^−/−^ (ns, *p* = 0.0962); *Xlf*^−/−^ vs. *Paxx*^−/−^ (ns, *p* > 0.9999); *Xlf*^−/−^ vs. *Dna-pkcs*^−/−^ (ns, *p* > 0.9999); *Xlf*^−/−^ vs. *Xlf*^−/−^*Paxx*^−/−^ (ns, *p* > 0.9999); *Xlf*^−/−^ vs. *Xlf*^−/−^*Dna-pkcs*^−/−^ (ns, *p* = 0.9081); *Paxx*^−/−^ vs. *Dna-pkcs*^−/−^ (ns, *p* > 0.9999); *Paxx*^−/−^ vs. *Xlf*^−/−^*Paxx*^−/−^ (ns, *p* > 0.9999); *Paxx*^−/−^ vs. *Xlf*^−/−^*Dna-pkcs*^−/−^ (ns, *p* = 0.2067); *Dna-pkcs*^−/−^ vs. *Xlf*^−/−^*Paxx*^−/−^ (ns, *p* > 0.9999); *Dna-pkcs*^−/−^ vs. *Xlf*^−/−^*Dna-pkcs*^−/−^ (ns, *p* > 0.9999); *Xlf*^−/−^*Paxx*^−/−^ vs. *Xlf*^−/−^*Dna-pkcs*^−/−^ (ns, *p* > 0.9999).

Detailed statistical analysis for the astrocyte differentiation, Figure 3D. WT vs. *Xlf*^−/−^ (ns, *p* > 0.9999); WT vs. *Paxx*^−/−^ (ns, *p* > 0.9999); WT vs. *Dna-pkcs*^−/−^ (ns, *p* > 0.9999); WT vs. *Xlf*^−/−^*Paxx*^−/−^ (ns, *p* > 0.9999); WT vs. *Xlf*^−/−^*Dna-pkcs*^−/−^ (ns, *p* > 0.9999); *Xlf*^−/−^ vs. *Paxx*^−/−^ (ns, *p* > 0.9999); *Xlf*^−/−^ vs. *Dna-pkcs*^−/−^ (ns, *p* > 0.9999); *Xlf*^−/−^ vs. *Xlf*^−/−^*Paxx*^−/−^ (ns, *p* = 0.0838); *Xlf*^−/−^ vs. *Xlf*^−/−^*Dna-pkcs*^−/−^ (ns, *p* > 0.9999); *Paxx*^−/−^ vs. *Dna-pkcs*^−/−^ (ns, *p* > 0.9999); *Paxx*^−/−^ vs. *Xlf*^−/−^*Paxx*^−/−^ (ns, *p* > 0.9999); *Paxx*^−/−^ vs. *Xlf*^−/−^*Dna-pkcs*^−/−^ (ns, *p* > 0.9999); *Dna-pkcs*^−/−^ vs. *Xlf*^−/−^*Paxx*^−/−^ (ns, *p* = 0.1392); *Dna-pkcs*^−/−^ vs. *Xlf*^−/−^*Dna-pkcs*^−/−^ (ns, *p* > 0.9999); *Xlf*^−/−^*Paxx*^−/−^ vs. *Xlf*^−/−^*Dna-pkcs*^−/−^ (ns, *p* = 0.5142).

Overall, XLF possesses functional redundancy with PAXX during the NSPC self-renewal, and with DNA-PKcs during cell growth and neuronal differentiation (Figure 2 and Figure 3).

## 4. Discussion

Here, we demonstrated that NHEJ factors XLF, PAXX and DNA-PKcs support proliferation of NSPCs during early mammalian neurogenesis, when the proliferation rate is high and the likelihood of DNA damages arising from DNA replication machinery is increased. In *Xrcc4*^−/−^, *Lig4*^−/−^, *Xlf*^−/−^*Paxx*^−/−^ and *Xlf*^−/−^*Dna-pkcs*^−/−^ mice NHEJ is ablated. Therefore, to avoid increased genomic instability during proliferation, developing neurons undergo programmed cell death via the p53-dependent pathway [2,9,10,16,17,19,20,27,28].

In particular, we found that proliferation of *Xlf*^−/−^, *Xlf*^−/−^*Paxx*^−/−^ and *Xlf*^−/−^*Dna-pkcs*^−/−^ NSPCs is significantly lower than that in WT cells; proliferation of *Xlf*^−/−^*Dna-pkcs*^−/−^ NSPCs is lower than that in *Paxx*^−/−^ cells (Figure 2B). However, the difference between the proliferation rates in XLF/PAXX or XLF/DNA-PKcs double-deficient NSPCs is not different from single deficient controls, and proliferation rates of PAXX- or DNA-PKcs-deficient NSPCs are not different from WT controls, suggesting that the effect is XLF-dependent and it is more prominent when two factors are inactivated (either XLF and PAXX or XLF and DNA-PKcs, Figure 2B). It was previously shown that the expression and activity of DNA-PKcs are increased in the NSPC murine cells when compared to mouse embryonic fibroblasts [26], suggesting that DNA-PKcs can be essential for homeostasis of NSPCs. Expression and activity of NHEJ vary in different cell types and require closer attention in future research [33]. Our data further highlighted this observation, and our findings suggest that the DNA-PKcs is required for NSPC proliferation and self-renewal capacities, although its role is partially compensated by XLF (Figure 2B,C).

Self-renewal capacity was significantly affected in NSPCs lacking XLF, PAXX or both XLF/PAXX, based on the cell number. However, inactivation of XLF or both XLF/DNA-PKcs did not change self-renewal of NSPCs when compared to WT controls (Figure 2C). Size of neurospheres is significantly reduced when XLF, DNA-PKcs or both XLF/DNA-PKcs are inactivated (Figure 2D). Moreover, lack of DNA-PKcs resulted in neurospheres of smaller size when compared to XLF- or PAXX-deficient samples (Figure 2D).

Finally, differentiation of NSPCs towards neurons or astrocytes was not significantly affected when the cells were lacking either XLF, DNA-PKcs, or PAXX (Figure 3). Nevertheless, we observed a trend towards reduced Tuj1+ neurons in cell populations lacking both XLF and DNA-PKcs (Figure 3A) when compared to WT and single-deficient controls. In addition, we observed a potential trend towards increased GFAP+ astrocytes in cell populations lacking either XLF, or PAXX, or both XLF and PAXX, when compared to WT controls (Figure 3B).

Overall, our data suggest that PAXX is required for self-renewal of NSPCs, while DNA-PKcs is essential for cell proliferation and growth of neurospheres (Figure 2B–D). None of the NHEJ proteins studied (XLF, DNA-PKcs, PAXX) was essential for the NSPC differentiation to neurons or astrocytes. Further analyses of early neurodevelopment in vivo and in vitro will help to reveal new insights regarding the role of NHEJ factors in neurodevelopment. Double- and multiple-knockout genetic models will facilitate these studies unraveling functional redundancy between the DNA repair factors.

Here, for the first time, we directly addressed the roles of NHEJ factors XLF, PAXX and DNA-PKcs in the processes of proliferation, self-renewal and differentiation of NSPCs. The roles of XLF and PAXX in neurodevelopment were previously characterized using murine embryonic brains, and the mouse embryos lacking both XLF and PAXX possessed increased neuronal apoptosis when compared to single-deficient and WT controls [16,17,19]. However, no previous studies involved NSPCs lacking XLF, PAXX or both XLF and PAXX. Furthermore, combined inactivation of XLF and DNA-PKcs resulted in ablated NHEJ and perinatal lethality of XLF/DNA-PKcs double deficient mice [27]. It was later suggested that the mice lacking both XLF and DNA-PKcs possess, among others, neurological defects [1,3]; however, no direct tests of this option were previously published. NSPC models were used to demonstrate that NHEJ factor XRCC4 as well as DNA damage response factor ataxia telangiectasia-mutated (ATM) are required for neuronal development, e.g., likely to repair DSBs, including ones in the long neural genes [34,35,36,37]. The DSB repair and DNA damage signaling were later more generally suggested to be required for healthy brain development, using macrocephaly and autism spectrum disorder-derived NSPCs [38]. Finally, it was recently demonstrated that regional regulator Filia is required for neurodevelopment. In this study, both murine hippocampus and the NSPCs’ research models were used [39].

Although mice lacking XLF possess normal CNS development [14,15], human patients with mutations in *Cernunnos*/*XLF* gene suffer from neurological defects, in addition to immunodeficiency [40,41]. The difference between human and murine phenotypes might be related to the fact that multiple NHEJ and DNA damage response factors, e.g., ATM and H2AX [42], MDC1 [43], 53BP1 [44,45], DNA-PKcs [27], PAXX [2,16,17,19,20], MRI [2,21] and RAG2 [46], partially compensate for the lack of XLF in mice. In other words, XLF compensates for the lack of multiple factors, including DNA-PKcs and PAXX. Our recent observations revealed that DNA-PKcs and PAXX are likely in the same sub-pathway of NHEJ because *Dna-pkcs*^−/−^*Paxx*^−/−^ mice and human cells do not possess any additional phenotype when compared to the *Dna-pkcs*^−/−^ or *Paxx*^−/−^ mice and cells [20,31]. In particular, human HAP1 cell lines lacking both DNA-PKcs/PAXX possess the same levels of genomic instability and sensitivity to DNA damage-inducing agents etoposide, doxorubicin and bleomycin as DNA-PKcs-deficient ones [20,31]. Moreover, mice lacking both DNA-PKcs and PAXX are live-born, fertile and do not show any additional phenotype when compared to immunodeficient *Dna-pkcs*^−/−^ knockout mice [20].

An important question challenging current research is: what is the mechanism underlying genetic interaction between *Xlf* and other NHEJ and DNA damage response (DDR) factors in cells? As one option, it was suggested that XLF can be complementary to the second factor having an alternative function (e.g., structural vs. enzymatic functions in XLF/ATM or XLF/DNA-PKcs double mutants). Alternatively, the proteins can be completely redundant key structural components of NHEJ machinery (in XLF/DNA-PKcs, XLF/MRI, XLF/PAXX, XLF/MDC1, XLF/53BP1, and XLF/RAG double mutants) [1,3,4]. Several models have been proposed to explain the DSB repair via NHEJ. A two-stage model, where DNA ends are first tied in a complex [47]. More specifically, first, Ku70-Ku80-DNA-PKcs (DNA-PK holoenzyme) form the initial complex, where DNA ends are sufficiently distant. Next, a larger complex is formed by DNA-PK, XLF, LIG4 and XRCC4 [47]. More recently, the XLF was proposed to be a flexible connector in this model, by interacting with both Ku70/Ku80 and XRCC4/LIG4/DSB [48].

Another model suggests that there are two major structural complexes formed during the NHEJ. Ku70-Ku80-XRCC4-LIG4 form the flexible synaptic complex (FS). Next, DSBs are brought together through interaction of XRCC4, giving rise to two Ku-XRCC4-Lig4-DNA complexes. Both XLF and PAXX are required for transition from the flexible synaptic to the second synaptic complex, or close synapsis (CS). Here, XLF stabilizes the close synapsis to a greater extent. In this model, it was suggested that DNA-PKcs is not involved in the FS and CS formation [49,50]. Moreover, the structural roles of MRI [21] and PAXX [19] were shown in the NHEJ, explaining the functional redundancy of these factors with XLF.

## 5. Conclusions

XLF is functionally redundant with PAXX during the neuronal stem and progenitor cells self-renewal and proliferation, and with DNA-PKcs during cell growth and neuronal differentiation. The NHEJ factors DNA-PKcs, PAXX and XLF are required for efficient early-stage development of neuronal stem and progenitor cells in mice. Additional NHEJ factors, such as MRI/Cyren, Ku70, Ku80, XRCC4 and LIG4, as well as multiple ATM-dependent DDR factors might have similar functions in neurodevelopment. Future studies will directly address the roles of NHEJ factors, including XLF, DNA-PKcs, PAXX and MRI, in learning, memory and mood regulations.

## Data Availability

The data presented in this study are available in Supplementary Materials.

## Supplementary Materials

The following are available online at https://www.mdpi.com/2218-273X/11/1/20/s1, Figure S1: Uncropped western blots for Figure 2A. Table S1: Commercial reagents; Table S2: Antibodies; Table S3: Equipment and Software; Table S4: Solutions and cell culture media; Table S5: Genotyping primers.
