# Supplementary material for: Non-Homologous End Joining Factors XLF, PAXX and DNA-PKcs Maintain the Neural Stem and Progenitor Cell Population
Source: Biomolecules. 2020 Dec 28;11(1):20. doi: 10.3390/biom11010020 (PMC7823790; doi:10.3390/biom11010020)
Supplement: Supplementary file 1 [file biomolecules-11-00020-s001.pdf]

Supplementary Figure 1 and Materials and Methods

Non-homologous end joining factors XLF, PAXX and DNA-PKcs are required to maintain the neural stem and progenitor cell population

Raquel Gago-Fuentes [1,2], and Valentyn Oksenych [1,3,4,*]

[1] Department for Cancer Research and Molecular Medicine (IKOM), Norwegian University of Science and Technology, Laboratory Center, Erling Skjalgssons gate 1, 7491 Trondheim, Norway;
[2] Department of Circulation and Medical Imaging, Norwegian University of Science and Technology, Prinsesse Kristinas gate 3, Akkutten og Hjertelunge-senteret, Postboks 8905, 7491 Trondheim, Norway;
[3] KG Jebsen Centre for B Cell Malignancies, Institute of Clinical Medicine, University of Oslo, N-0316 Oslo, Norway;
[4] Institute of Clinical Medicine, University of Oslo, 0318 Oslo, Norway; valentyn.oksenych@medisin.uio.no
* Correspondence: valentyn.oksenych@medisin.uio.no (V.O.)

Key words: DNA repair; NHEJ; synthetic lethality; genetic interaction

* Corresponding authors:

valentyn.oksenych@medisin.uio.no (Valentyn Oksenych)

Supplementary Figure S1. Uncropped western blots

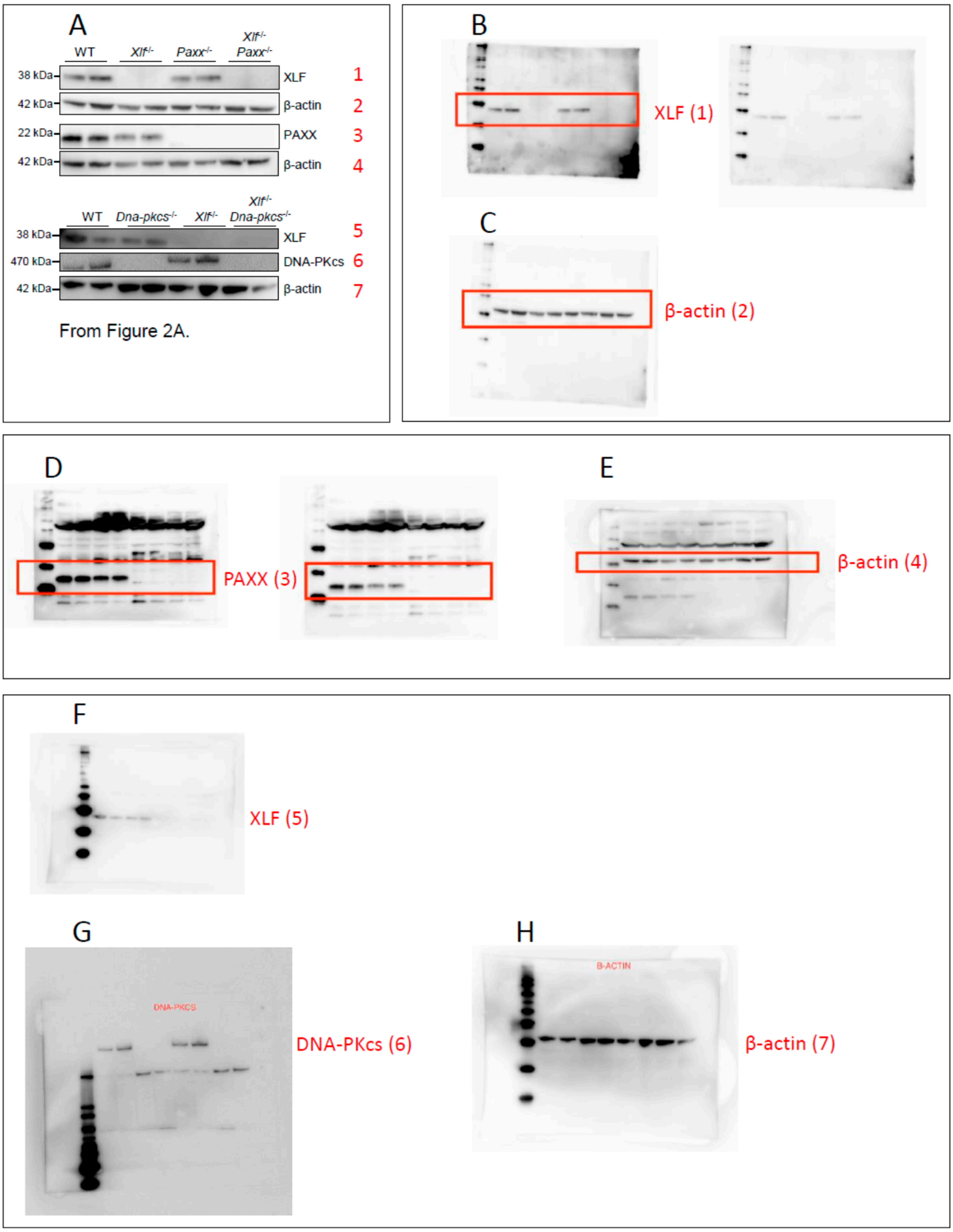

Supplementary Figure S1. Uncropped western blots for Figure 2A.
(A) Original Figure 2A. 1-7 indicate western blot numbers, uncropped images are presented in B-H.
(B) Western blot detecting XLF (1). Longer (left) and shorter (right) exposures are provided.
(C) Western blot detecting a loading control for XLF gel (1), β-actin (2).
(D) Western blot detecting PAXX (3). Longer (left) and shorter (right) exposures are presented.
(E) Western blot detecting a loading control for PAXX gel (3), β-actin (4).
(F) Western blot detecting XLF (5).
(G) Western blot detecting DNA-PKcs (6).
(H) Western blot detecting a loading control for XLF (5) and DNA-PKcs (6) gel, β-actin (7).

Supplementary Table S1. Commercial reagents

Reagent	Catalogue number, Company, Country
Proteinase K	#1703001, Invitrogen, Carlsbad, CA, USA
Trizma	#T3253, Sigma, St. Louis, MO, USA
KCl	#P9541, Sigma, St. Louis, MO, USA
NP-40	#127087-87-0, Sigma, St. Louis, MO, USA
Tween-20	#9005-64-5, Sigma, St. Louis, MO, USA
GoTaq®G2 Green Master Mix	# M7823, Promega, Madison, WI, USA
DMEM/F12 Medium	#11330-057,Thermo Fisher Scientific, Waltham, MA, USA
Penicillin/Streptomicyn	#15140-122, Thermo Fisher Scientific, Waltham, MA, USA
B27 supplement	#17504044, Thermo Fisher Scientific, Waltham, MA, USA
Epidermal Growth Factor (EFG)	# AF-100-15, PeproTrech, Sweden
basic-Fibroblast Growth Factor (b-FGF)	#100-18B, PeproTech, Sweden
Trypsin-EDTA 0.25 %	#T3924, Sigma, St. Louis, MO, USA
PrestoBlue™ Cell Viability Reagent	#A13262, Invitrogen, Carlsbad, CA, USA
Neurobasal A Medium	#10888-022, Thermo Fisher Scientific, Waltham, MA, USA
Poly-D-lysine	# p0899, Sigma, St. Louis, MO, USA
Laminin	# L2020, Sigma, St. Louis, MO, USA
B27 supplement without vitamin A	#12587010, Thermo Fisher Scientific, Waltham, MA, USA
GlutaMAX	#35050-038, Thermo Fisher Scientific, Waltham, MA, USA
Triton X-100	#T8787, Sigma, St. Louis, MO, USA
Bovine serum albumin (BSA)	#A2153, Sigma, St. Louis, MO, USA
Goat antiserum	#10000C, Invitrogen, Carlsbad, CA, USA
4´6-diamidino-2-phenylindole (DAPI)	#62248, Molecular Probes, Eugene, OR, USA
RIPA	#R0278, Sigma, St. Louis, MI, USA
cOmplete™ EDTA-free Protease Inhibitor	#11873580001, Roche, USA
Phenylmethane sulfonyl fluoride (PMSF)	#70137720, Sigma, St. Louis, MI, USA
Bradford reagent	#5000006, BioRad, Hercules, CA, USA
Phosphate-Buffered Saline (PBS)	#BR0014G, Oxoid Limited, Hampshire, UK
20x NuPAGE Transfer Buffer	#NP0006-1, Life Technologies, Carlsbad, CA, USA
SuperSignal™ West Femto	#34095, Thermo Fisher Scientific, Waltham, MA, USA

Supplementary Table 2. Antibodies

Antibody	Catalogue number, Dilution, Company, Country
Mouse anti-neuron specific β-tubulin (Tuj1)	#MAB1195, 1:600, R&D Systems, USA
Mouse anti-glial fibrillary acid protein (GFAP)	#G3893, 1:600, Sigma, USA
Rabbit anti-glial fibrillary acid protein (GFAP)	#Z0334, 1:1000, Dako, Denmark
Goat anti-mouse Alexa 488	#A11001, 1:500, Molecular Probes, USA
Goat anti-rabbit Alexa 594	#A11037, 1:500, Molecular Probes, USA
Rabbit anti-XLF	#A300-730A , 1:1000, Bethyl, USA
Rabbit anti-C9orf142 (PAXX)	#126353, 1:200, Novus Biologicals, USA
Mouse anti-DNA-PKCS	#MA5-13404, 1:1000, Invitrogen, Carlsbad, USA
Mouse anti-β-actin	#Ab8226, 1:2000, Abcam, UK
Swine anti-rabbit	#P0399, 1:2000, Dako, Denmark
Goat anti-mouse	#P0447, 1:2000, Dako. Denmark

Supplementary Table 3. Equipment and software

Equipment, software	Company, Country
FLUOstar Omega	BMG Labtech, Ortenberg, Germany
EVOS microscope	Invitrogen, Carlsbad, USA
ChemiDoc™ Touch Imaging System	BioRad, Hercules, USA
ImageJ	National Institute of Health, Bethesda, USA
GradhPad Prism software	GradhPad Prism, La Jolla, CA, USA

Supplementary Table 4. Solutions and cell culture media

Solution, medium	Composition
DNA lysis solution	10 mM pH 9 Trizma, 1 M KCl, 0.4% NP-40 and 0.1% Tween20
Proliferation medium	DMEM/F12 medium supplemented with 1% penicillin/streptomycin, 2% B27 without vitamin A, 10 ng/ml EGF and 20 ng/ml bFGF
Differentiation medium	NeuroBasal A medium supplemented with 1% penicillin/streptomycin, 2% B27, 1 % GlutaMAX and 10 ng/ml bFGF
Blocking solution (10x)	10% BSA (*Sigma, USA*), 10% goat serum and 0.1% Triton X-100
PBST	10% Tween20 in PBS

Supplementary Table 5. Genotyping primers

Gene	Sequence
Xlf wild type (650 bp)	*Forward: CATGTTGGCTCTGCGAATAGA*<br>*Reverse: GAGCTCGGATATGAGCGCTCAG*
Xlf knockou*t* (950 bp)	*Forward: CTGTCTTGTGGGCATAGTAGGC*<br>*Reverse: GAGCTCGGATATGAGCGCTCAG*
Paxx (965 bp wild type; 298, 312, 329 bp knockout)	*Forward: ACAGAGGGTGGTGACTCAGACAATGG*<br>*Reverse: GGAAATGCTATTAGAACCACTGCCACG*
Dna-pkcs wild type (250 bp)	*Dnapkcs-1: CCCTCCAGACAGCCAGCTAAGACAGG*<br>*Dnapkcs-2: GAAAAAGTCTATGAGCTCCTGGGAG*
Dna-pkcs knockout (427 bp)	*Dnapkcs-1: CCCTCCAGACAGCCAGCTAAGACAGG*<br>*Dnapkcs-3: ACGTAACTCCTCTTCAGACCT*
Trp53 wild type (321 pb)	*Trp53-1: TGGATGGTGGTATACTCAGAGC*<br>*Trp53-2: AGGCTTAGAGGTGCAAGCTG*
Trp53 knockout (110 bp)	*Trp53-1: TGGATGGTGGTATACTCAGAGC*<br>*Trp53-3: CAGCCTCTGTTCCACATACACT*
